# Differential Repeat Accumulation in the Bimodal Karyotype of *Agave* L.

**DOI:** 10.3390/genes14020491

**Published:** 2023-02-15

**Authors:** Lamonier Chaves Ramos, Mariana Báez, Joerg Fuchs, Andreas Houben, Reginaldo Carvalho, Andrea Pedrosa-Harand

**Affiliations:** 1 Laboratory of Plant Cytogenetics, Graduate Program in Agronomy, Genetic Plant Breeding—PPGAMGP, Department of Agronomy, Federal Rural University of Pernambuco, Recife 52171-900, Brazil; 2 Laboratory of Plant Cytogenetics and Evolution, Department of Botany, Federal University of Pernambuco, Recife 50670-420, Brazil; 3 Plant Breeding Department, University of Bonn, Katzenburgweg 5, 53115 Bonn, Germany; 4 Leibniz Institute of Plant Genetics and Crop Plant Research (IPK), 06466 Stadt Seeland, Germany

**Keywords:** bimodality, centromeric satellite, hybrid, macro- and microchromosomes, repetitive DNA, sisal, satellite DNA, transposable elements

## Abstract

The genus *Agave* presents a bimodal karyotype with *x* = 30 (5L, large, +25S, small chromosomes). Bimodality within this genus is generally attributed to allopolyploidy in the ancestral form of Agavoideae. However, alternative mechanisms, such as the preferential accumulation of repetitive elements at the macrochromosomes, could also be important. Aiming to understand the role of repetitive DNA within the bimodal karyotype of *Agave*, genomic DNA from the commercial hybrid 11648 (2*n* = 2*x* = 60, 6.31 Gbp) was sequenced at low coverage, and the repetitive fraction was characterized. In silico analysis showed that ~67.6% of the genome is mainly composed of different LTR retrotransposon lineages and one satellite DNA family (AgSAT171). The satellite DNA localized at the centromeric regions of all chromosomes; however, stronger signals were observed for 20 of the macro- and microchromosomes. All transposable elements showed a dispersed distribution, but not uniform across the length of the chromosomes. Different distribution patterns were observed for different TE lineages, with larger accumulation at the macrochromosomes. The data indicate the differential accumulation of LTR retrotransposon lineages at the macrochromosomes, probably contributing to the bimodality. Nevertheless, the differential accumulation of the satDNA in one group of macro- and microchromosomes possibly reflects the hybrid origin of this *Agave* accession.

## 1. Introduction

A bimodal karyotype is composed of two sets of chromosomes of contrasting sizes, known as macro- (large) and microchromosomes (small). These karyotypes have, in general, an old origin, and their conservation may be related to some adaptive advantage [1] and/or mechanism of karyotypic orthoselection, as suggested for species of the genus *Hippeastrum* Herb. [2]. The origin of bimodal karyotypes could be attributed to three possible mechanisms. First, these karyotypes could be the consequence of chromosome rearrangements involving fusion–fission events, where macrochromosomes are the products of microchromosome fusions [3], or these are the results of macrochromosome fission, as observed in some bimodal karyotypes of the Lilieae tribe (Liliaceae) [4]. Second, allopolyploidy could result in a bimodal karyotype by the hybridization of parent species with different chromosome sizes, confirmed in *Aloe* L. (Asphodelaceae) [5] and *Milium montianum* Parl. (Poaceae) [6]. Third, the differential accumulation of repetitive DNA sequences has contributed to the increased size of a chromosome subset within the bimodal karyotypes of *Muscari comosum* (L.) Miller (Hyacinthaceae) [7], *Eleutherine bulbosa* (Miller) Urban (Iridaceae) [8] and *Cuscuta* L. bimodal species [9].

Repetitive sequences are involved in genome evolution, causing differences in DNA content between species because of insertions and deletions of repeats [10,11]. In plants, transposable elements (TEs) and satellite DNAs (satDNAs), as well as ribosomal DNAs, are the most abundant, contributing to karyotype evolution [12,13]. Next-Generation Sequencing (NGS) has allowed detailed analyses of the composition of the genomes, especially the characterization of repetitive sequences and comparison of their evolutionary dynamics [9,14,15], including their dynamics in allopolyploid genomes [10,16].

The genus *Agave* L. (Asparagaceae Juss.) is characterized by having a highly conserved bimodal karyotype. Its monoploid set generally consists of 5 macrochromosomes and 25 microchromosomes, with some variation especially at higher ploidy levels [17,18]. Bimodality in this genus was attributed to allopolyploidy, although the possibility of chromosomal fusion and fission events could not be discounted [19]. The genus has different ploidy levels, with species ranging from diploid to octoploid [20]. *Agave sensu lato*, including *Manfreda*, *Polianthes*, and *Prochnyanthes*, is endemic to America, and comprises around 200 species. It is monocarpic, which means that the plant reproduces only once after many years and then dies [21]. Some *Agave* species are grown to produce natural fibres as a sustainable alternative for industrial products [22]. Because of their slow growth rate, long lifespan (8 to 20 years) and inefficient sexual reproduction, it is very difficult to genetically improve agaves, and consequently, little has been achieved. Most of the species of the genus are wild and constitute an important genetic resource for breeding purposes [17]. There is only one example of a breeding program implemented in today’s Tanzania in the early 20th century, which produced the only *Agave* hybrid (H11648) ever commercially exploited [23]. Agronomically, this hybrid stands out for its drought tolerance, significant leaf formation potential and superior fibre production per hectare, being the main one among the few genotypes widely cultivated for this purpose worldwide [24].

The H11648 cultivar has a diploid chromosome number of 2*n* = 2*x* = 60, with a genome size of 1C = 7.61 pg [25,26], and is originally from Africa, obtained through a backcross between *Agave angustifolia* Haw. × *Agave amaniensis* Trel. and Nowell and *A. amaniensis* [23,27,28]. The hybrid is fertile, producing viable seeds, although it is usually clonally propagated. Both parental species possess 2*n* = 60 chromosomes with similar karyotypes [17,29,30], although *A. amaniensis* is not investigated in detail. Tetraploid and hexaploid varieties of *A. angustifolia* are also known [26], and intraspecific variation between diploid *A. angustifolia* cultivars was reported, with differences in the karyotype formulae (for instance, 42 m + 4 sm + 6 st + 8 t for “Cimarron” and 48 m + 2 sm + 2 st + 8 t for “Lineño”) and polymorphism of the secondary constriction, among other cytogenetic parameters [17,30]. One locus of 5S and 35S rDNA is present per basic, monoploid genome. The 5S rDNA locate proximally on a small submetacentric chromosome and the 35S rDNA is located in the secondary constriction of a large acrocentric chromosome, as observed for *A. angustifolia* and other *Agave* species [26,31]. Phylogenetically, *A. angustifolia* and *A. amaniensis* are closely related to each other and to *A. americana*, and the position of the hybrid H11648 in the same clade corroborates its known origin [32,33].

In order to characterize the repeats of *Agave*, the repetitive fraction of the *Agave* hybrid 11648 was in silico analysed, and the chromosomal distribution of the most abundant sequences was investigated. We selected H11648 because this accession is diploid and shows a typical *Agave* bimodal karyotype. In addition, this accession is cultivated worldwide due to its economic importance and is readily available. The characterization of its genome could assist further breeding. If bimodality is solely associated with allopolyploidy, we expected distinct repetitive elements from the ancestral species in either the macro- or microchromosomes. In the case of dysploidy by chromosome fusion, the distribution of repeats in multiple blocks along the macrochromosomes could be indicative of microchromosome fusions. However, the enrichment of repetitive sequences in macrochromosomes could only suggest that bimodality was correlated to the differential accumulation of repeats in macrochromosomes.

## 2. Materials and Methods

### 2.1. Plant Material

Young clones and seeds of the *Agave* hybrid H11648 were provided by the Brazilian Agricultural Research Corporation Embrapa-Cotton, Paraíba, Brazil. Genomic DNA was extracted from seedlings, which was also used for estimating the genome size. Asexually reproduced individuals were kept in pots under external environmental conditions (approx. 30 °C and high humidity) in the experimental garden of the Laboratory of Plant Cytogenetics and Evolution, Federal University of Pernambuco, Brazil, and used to collect roots for cytological preparations.

### 2.2. Slide Preparation and Double Staining with CMA/DAPI

Root tips were pre-treated with 2 mM 8-hydroxyquinoline for 24 h at 10 °C, fixed in methanol: acetic acid (3:1, *v*/*v*) for 2 h at room temperature and stored at −20 °C. After washing in distilled water, root tips were digested in a citrate phosphate buffer solution containing 2% cellulose (*w*/*v*, Onozuka), 20% pectinase (*v*/*v*, Sigma, St. Louis, MO, USA) at 37 °C for 3 h. After enzymatic digestion, the root tips were disintegrated, with the aid of needles, in a cold fixative (methanol: acetic acid 3:1, *v*/*v*). With the use of an air pump, the meristem was spread over the surface of a slightly tilted slide. Then, slides were dipped in 45% acetic acid for 30 s and taken to a flat, preheated surface until completely dried at 37 °C, modified from [34].

Staining with chromomycin A3 (CMA) and 4’,6-diamidino-2-phenylindole (DAPI) fluorochromes was performed according to [35]. Slides were aged for three days at room temperature, stained with CMA at 0.1 mg/mL in McIlvaine buffer for 60 min and with 2 µg/mL DAPI for 30 min, and mounted in McIlvaine-glycerol buffer 1:1 (*v*/*v*). After image acquisition, the slides were destained with ethanol: acetic acid (3:1, *v*/*v*) for 30 min, then dehydrated with ethanol for 60 min at room temperature, air-dried and used for sequential fluorescent in situ hybridization with ribosomal DNA probes.

### 2.3. Genome Size Estimation

Samples were prepared from 40 to 50 mg of young leaves in 1 mL of LB nuclear isolation buffer and filtered through a 30 µm nylon filter [36]. *Hordeum vulgare* L. (2C = 16.01 pg) served as standard. Nuclei were stained with propidium iodide (50 µg/mL), and RNase A (50 mg/mL) was added to prevent staining of RNA. The nuclear DNA content was determined with a Partec CyFlow SL (Partec) flow cytometer, and results were analysed with the Flomax program. For genome size estimations, three replicates were analysed, in two different days, and the nuclear DNA content of *Agave* H11648 was calculated according to [36].

### 2.4. Sequencing of Genomic DNA and Repeat Characterization

Genomic DNA was extracted from young leaves using the DNAeasy Plant MiniKit kit (QIAGEN) following the manufacturer’s recommendations and sequenced through the Illumina platform at IPK, Gatersleben, Germany, generating paired-end reads of 101 bp of length (GenBank Bioproject PRJNA934096). The obtained reads were used to identify and characterize the most abundant repetitive DNA families using the *RepeatExplorer* platform (http://repeatexplorer.umbr.cas.cz, accessed on 19 December 2016) [37,38]. This pipeline groups sequences by similarity, generating clusters for different repetitive DNA families. Standard parameters were used, and the clustering was performed with a minimum overlap of 55% and a similarity of 90% throughout the sequence. Protein domains were identified using the Find RT Domains tool within the platform. The clusters were identified and classified through searches in various databases, as well as by similarity searches (BLASTn and BLASTx) against GenBank and TIGR BLAST search. The layouts of the graphs of individual clusters were examined using the SeqGrapheR program [38]. Clusters containing satellite DNA were identified based on the presence of tandem sub repetitions within their sequences or assembled contigs using DOTTER [39]. All families of repetitive sequences were annotated manually, using the results of the different programs. Mobile elements were named according to [40].

### 2.5. Sequence Amplification and Fluorescent In Situ Hybridization (FISH)

Primers for the six most abundant sequences of transposable elements (LTR retrotransposons Ty1/copia SIRE, Tork and TAR, and LTR retrotransposons Ty3/gypsy Ogre, Athila and Chromovirus) were designed based on the Integrase domain using the Geneious 7.0 software (Supplementary Table S1). For the satellite DNA family, primers were designed facing outwards, anchored at the most conserved region of the monomer, using the same software. The repetitive sequences were amplified by PCR under the following conditions: initial denaturation at 94 °C for 3 min, followed by 30 amplification cycles, each consisting of 94 °C for 1 min, 60 °C for 1 min and 72 °C for 1 min, and 10 min final elongation at 72 °C. PCR reactions included 50 ng genomic DNA, 0.5 μM of each primer, 0.1 mM dNTP, 1× PCR buffer (Invitrogen, Waltham, MA, USA), 2 mM MgCl_2_, 1× TBT (50 mM trehalose, 1 mg/mL BSA, 1% Tween 20 and 8.5 mM Tris hydrochloride) and 0.04 U of Taq Polymerase (Invitrogen). Products were checked on a 1% agarose gel, and sequences were confirmed and labelled with Cy3-dUTP (GE) by nick translation (Roche, Basel, Switzerland).

To locate the ribosomal DNA sites, a clone containing a 500 bp fragment of the 5S rDNA of *Lotus japonicus* (Regel) K.Larsen (Fabaceae) [41] was labelled with Cy3-dUTP (GE), and a clone containing a 6.5 kb fragment corresponding to the 35S rDNA of *Arabidopsis thaliana* [42] was labelled with digoxigenin-11-dUTP (Roche). The 35S rDNA probe was detected with anti-digoxigenin produced in sheep, conjugated with FITC (Roche), and signals were amplified with anti-sheep IgG produced in rabbits also conjugated with FITC (Serotec).

FISH was performed according to [41], with minor modifications. The hybridization mix was composed of 50% formamide (*v*/*v*), 10% dextran sulphate (*w*/*v*), 2× SSC and 50 ng/μL of each probe. The slides were denatured at 75 °C for 3 min, and the final stringency of hybridization was 76%.

### 2.6. Image Acquisition and Processing

The images were captured with a Leica DM5500B microscope coupled with a Leica DFC345 FX camera and the LAS AF software. The images were uniformly optimized for brightness and contrast using Adobe Photoshop CS3.

## 3. Results

### 3.1. Identification and In Silico Characterization of Repetitive Sequences

Eight million 101 bp long sequence reads were obtained (800 Mbp) and, considering the genome size of the hybrid *Agave* H11648 2C = 12.91 pg (or 1C = 6.31 Gbp), this amount of reads corresponded to a genome coverage of approximately 0.13×. The automatic subsampling of 33.7% of the total input reads in the *RepeatExplorer* platform generated 43,944 clusters, containing 2 to 55,804 reads each. Of this total, 268 clusters containing at least 0.01% of the genome were annotated.

After annotation of the clusters and elimination of plastidial and mitochondrial DNA sequences, the analysis revealed that ~67.2% of the nuclear genome is composed of repetitive DNA. A total of 99% of repeats were classified, and 2% of the nuclear DNA is composed of a single satellite DNA family called AgSAT171, with a monomer length of ~171 bp. The most abundant elements were LTR retrotransposons, which constituted 62.5% of the nuclear DNA. Around 25% were classified as Ty1/copia elements, with the most abundant lineages being SIRE (4%), Tork (1.92%) and TAR (1.41%). Of the elements, 16.6% were annotated as Ty3/gypsy, with the most abundant lineages being Ogre, Athila and Chromovirus with 3.36%, 2.37% and 1.97%, respectively (Table 1).

### 3.2. Differential Distribution of Repeats along Micro- and Macrochromosomes

**Figure 1:**
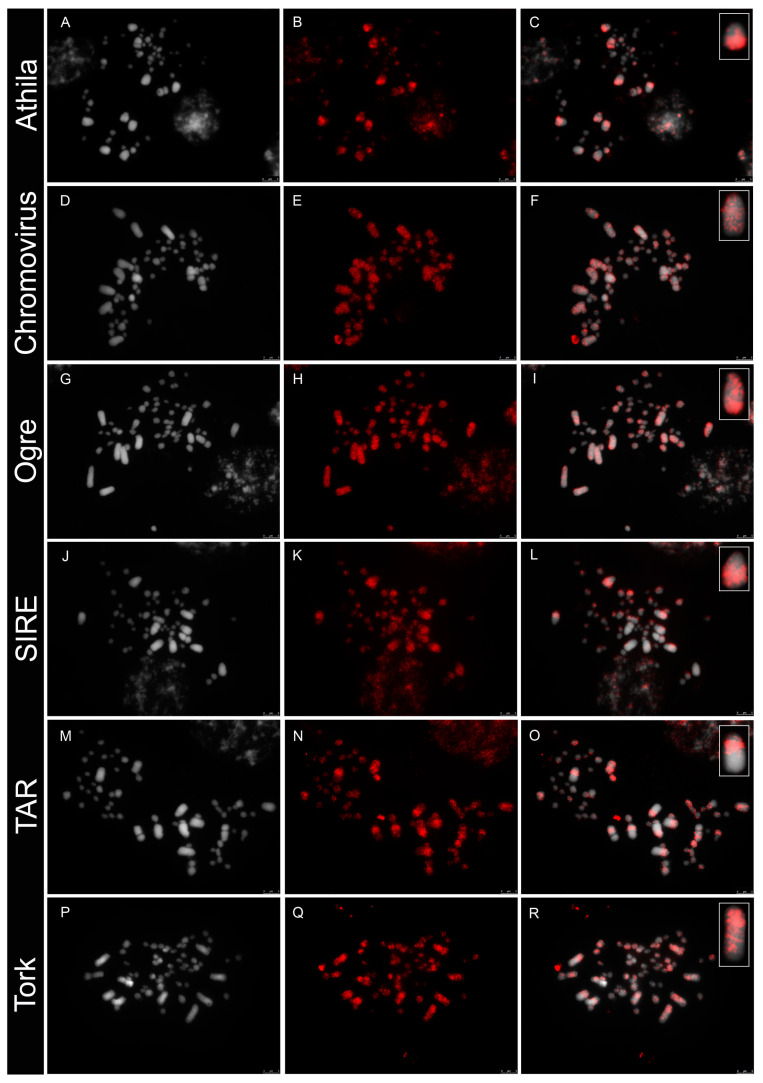
Distribution of the most abundant LTR retrotransposons (in red) in the mitotic chromosomes of hybrid *Agave* 11648 (DAPI in grey) after FISH, showing the differential chromosome patterns and accumulation at the macrochromosome pairs. (**A**–**C**), Ty3/gypsy Athila; (**D**–**F**), Ty3/gypsy Chromovirus; (**G**–**I**), Ty3/gypsy Ogre; (**J**–**L**), Ty1/copia SIRE; (**M**–**O**), Ty1/copia TAR; (**P**–**R**), Ty1/copia Tork. Insets show one macrochromosome of the same cell at higher magnification.

In order to understand the distribution of the main repetitive sequences in the bimodal karyotype of *Agave*, the six most abundant TEs (three Ty1/copia and three Ty3/gypsy elements) were amplified by PCR, labelled directly and used as probes for fluorescent in situ hybridization to mitotic chromosomes. All transposable elements showed a dispersed distribution, both in the micro- and macrochromosomes. However, some elements were differentially enriched in specific regions. The Ty1/copia Tork and TAR lineages were mainly distributed at proximal regions of the long arms of the macrochromosomes, while Ty1/copia SIRE and Ty3/gypsy Athila lineages were located more densely dispersed at interstitial and distal regions. On the other hand, Ty3/gypsy Chromovirus and Ogre lineages showed a dispersed distribution, with no particular intense region, along the macrochromosomes. All elements probed showed a dispersed labelling along microchromosomes, with variable intensities, but signals were generally weaker than along macrochromosomes (Figure 1).

**Figure 2:**
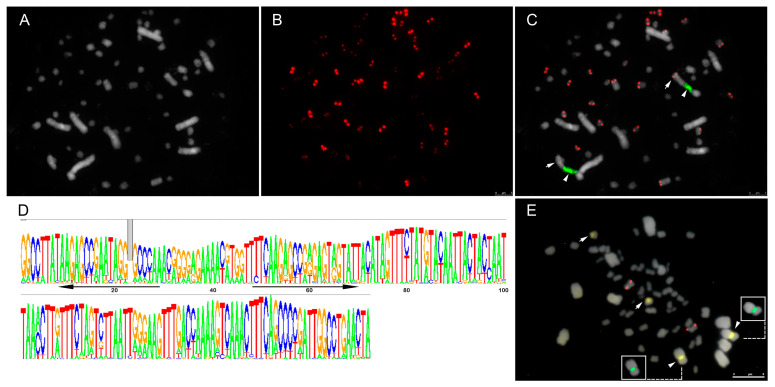
Distribution of tandem repeats and heterochromatin on chromosomes of *Agave* hybrid H11648. (**A**) DAPI (in grey); (**B**) AgSAT171 probe (in red) showing strong or weak signals in all chromosomes; (**C**) overlay of AgSAT171 (red) and 35S rDNA site (green, arrowheads) showing 20 chromosomes with stronger signals and heteromorphism for the chromosome pair bearing the rDNA site; (**D**) consensus sequence logo of AgSAT171 (black arrows signalized the primer positions); (**E**) CMA^+^/DAPI^−^ heterochromatic bands (yellow); 5S (red) and 35S ribosomal DNA (in green, insets), with the 35S colocalizing with CMA^+^ bands (arrowheads). Arrows indicate the difference in AgSAT171 signal intensity between chromosomes of the large pair that harbours the 35S rDNA site (in **C**) or the second CMA^+^ block that does not colocalize with the rDNA (in **E**).

The only satellite, AgSAT171, showed a single pair of dot-like signals per chromosome, associated with the primary constriction, when visible. However, strong signals were observed in only 20 chromosomes of the complement, 1 macro- and 19 microchromosomes, suggesting an enrichment of this repeat in a subset of macro- and microchromosomes (Figure 2). Double CMA/DAPI staining was used to visualize regions associated with heterochromatin and, together with the 35S and 5S rDNA sites, allowed the identification of three chromosome pairs. A pair of macrochromosomes had a CMA^+^/DAPI^−^ interstitial band, colocalized with the 35S rDNA site. A pair of microchromosomes was also evidenced by a CMA^+^/DAPI^−^ band, and a different microchromosome pair presented the 5S rDNA site (Figure 2). Single chromosome pairs bearing rDNA sites is a common feature among *Agave* species [26,31]. In the 35S rDNA carrying chromosome pair, the difference in intensity of the putative centromeric AgSAT171 signal between both chromosomes was very evident (arrows in Figure 2).

Thus, the chromosomal distribution of the main repetitive sequences of the *Agave* genome showed an accumulation of LTR retroelements, both Ty1/copia and Ty3/gypsy, in the macrochromosomes. On the other hand, tandem repeats were similarly distributed in both macro- and microchromosomes.

## 4. Discussion

We characterized the repetitive fraction of a representative of the *Agave* genus and revealed that most of its genome (~67.6%) is composed of different families of repeats, 62.5% being LTR retroelements, compatible with its relatively large genome (1C = 6.31 Gbp), although slightly smaller than previously reported (7.61 pg) [26]. This estimate of repeat content is much higher than that proposed for *Hosta* Tratt. from the same subfamily Agavoideae (Asparagaceae), with a repetitive DNA fraction of 4.49% and a genome size of 1C = 19.12 Gbp. At least part of this difference might be due to the different methodologies used [43]. In our analysis, Ty1/copia were the most abundant and diversified elements, as also observed for *Agave tequilana* Weber, for which a detailed molecular characterization of Ty1/copia elements is provided [44].

Despite the high abundance of repeats in general, only one family of satellite DNA with a genome abundance above 0.01% was found, which shared no similarity with a previously characterized satellite-like DNA from *A. angustifolia* Haworth, *A. tequilana* Webber and *A. fourcroydes* Lemaire [45]. The high proportion of retroelements and the low proportion of satellite DNA may explain the small amount of heterochromatin observed by CMA/DAPI staining, despite its large genome size. A small amount of satDNA was also observed in other plant species with larger genomes, such as *Passiflora quadrangularis,* with the largest genome known for its genus and only a few satellites, mostly related to the 35S rDNA [46]. Nevertheless, LTR-TEs may also form CMA^+^ bands in chromosomes, such as in some legumes from the Caesalpinia group where CMA^+^ bands are composed mainly of the Ty3/gypsy Tekay lineage [47] and several species from the *Phaseolus* genus with a complex composition involving different repetitive sequences [48]. Similarly, CMA/DAPI may not reveal large satDNA blocks if they are not particularly enriched in A/T or G/C, as seen in the Brazilian species of *Alstroemeria* genus with some satellites located on euchromatin [14].

Centromeric satellite DNA is commonly the most abundant satDNA of a species [49], and, indeed, the only abundant satellite DNA family of *Agave* is a putative centromeric repeat, based on its chromosomal distribution. The large *Agave* chromosomes are subtelocentric or telocentric [17,22], which is congruent with the position of AgSAT171. In addition to the 35S rDNA site, which colocalized with a CMA^+^/DAPI^−^ band, a second heterochromatic band was observed in the genome, suggesting the existence of at least a second (not identified) tandem repeat of low abundance. Additional approaches [50] could be employed for identifying low-abundant satDNAs in *Agave*.

The centromeric satDNA varied in signal intensity between chromosome pairs, with a stronger signal in one-third of the chromosomes, suggesting that they contain significantly more copies of the AgSAT171 repeat. However, this distribution is not related to the bimodal condition of this karyotype, since both macro- and microchromosomes showed strong and weak hybridization signals. The difference in the abundance of centromeric satellite DNA among chromosomes may be related to the fact that the genotype originated from an interspecific backcross. In *Saccharum spontaneum*, an octoploid species, the distribution of centromeric satDNA also revealed high-intensity signals in only 8 of the 64 chromosomes of the complement; however, in this case, this observation was related to the ploidy level of species [51]. In the case of *Agave*, our hypothesis is that *A. angustifolia* presents a greater abundance of AgSAT171 than *A. amaniensis*. This latter species is the recurrent parent in the crossings that originated this *Agave* hybrid, suggesting that the two-thirds of chromosomes which have less AgSAT171 are coming from *A. amaniensis*. If confirmed, this repeat could be used as a marker to assist breeding programs for *Agave* hybrids. 

Different lineages of retroelements displayed a differential distribution along the macrochromosomes. Ty1/copia Tork and TAR lineages were enriched at proximal regions of the long arms, while Ty1/copia SIRE and Ty3/gypsy Athila were enriched at interstitial and distal regions of the same chromosome arms. In most of the species with small genomes, the distribution of these elements is frequently related to the pericentromeric region, as seen in species from *Phaseolus* [48] and *Passiflora* [46]. On the other hand, species with larger genomes present a uniform distribution of most retroelements, commonly without any specific pattern along the chromosomes, as observed in the larger genomes within the *Passiflora* subgenus [46]. In different bimodal karyotypes, a combination of these patterns with the large chromosomes showing a dispersed uniform distribution and the small chromosomes an enrichment at pericentromeric regions was found, such as in *E. bulbosa* [8] and some species of *Cuscuta* [9]. Here, in addition to presenting differential dispersed distribution between chromosome sets, the largest chromosomes of the *Agave* bimodal karyotype display specific patterns for different lineages of repetitive sequences along the chromosomes.

Since the karyotype with 5 macro- and 25 microchromosomes pairs was first described [52], both fusion and fission and, more recently, allopolyploidy have been proposed as mechanisms to explain the bimodality [18,19]. Karyotype comparisons involving several species of *Agave* and closely related groups showed that the genus *Agave* has a smaller number of macrochromosomes and an increase in the number of microchromosomes, in addition to acro/telocentric chromosomes, suggesting fission events [18]. On the other hand, the analysis of transcriptome data indicated events of whole-genome duplication (WGD), with one WGD shared between species with bimodal karyotype, suggesting that the origin of the bimodal karyotype in the subfamily Agavoideae may be related to allopolyploidy [19]. The distribution of repetitive sequences on the chromosomes of *Agave* does not support any of these hypotheses for the origin of chromosomal bimodality in Agavoideae but provides support for the contribution of multiple LTR retrotransposons to the size of macrochromosomes within this group.

Different lineages of LTR retrotransposons accumulated in more significant proportion in the macrochromosomes of *Agave*, showing similarity to that observed in *E. bulbosa*. In this species, which has one pair of macrochromosomes and five pairs of microchromosomes, it was seen that both satellite DNA and several LTR retrotransposon probes showed more intense and uniform labelling in the larger chromosome pair after FISH [8]. In *Agave*, three of these same lineages (Tork, SIRE and Chromovirus) were more abundant in macrochromosomes. However, instead of a uniform distribution for all of them, some were preferentially enriched in the proximal regions and others in the more distal regions of the *Agave* macrochromosomes. In addition, even the microchromosomes showed a relatively uniform distribution for the same elements, instead of a pericentromeric distribution, as observed for the microchromosomes of *Eleutherine* and in small genome species. Therefore, *Agave* repeats have an unusual chromosomal distribution of repeats, perhaps related to its bimodal organization.

## 5. Conclusions

Altogether, considering the distribution of the observed repeats, the most likely scenario is that a differential accumulation of different repetitive sequences has contributed to the size increase of macrochromosomes in *Agave*. However, considering an ancestral allopolyploidy event, it is possible that the ancient origin of the group’s bimodal karyotype led to a homogenization of sequences between the two sets of chromosomes, without changing, in this case, the bimodal organization of these karyotypes.

## Figures and Tables

**Table 1:** Genome proportion of major repetitive elements of the *Agave* hybrid 11648.

Repetitive Elements		Genome %
Retrotransposons		64.32
	SINE	0.46
LTRs		62.50
LTR non classified		20.99
Ty1/copia		24.90
	SIRE	4.00
	Tork	1.92
	TAR	1.41
	Angela	0.90
	Bianca	0.17
	Ivana/Oryco	0.16
	Ale I	0.12
	Ale II	0.12
	Unclassified Ty1/copia	16.12
Ty3/gypsy		16.61
	Ogre	3.36
	Athila	2.37
	Chromovirus	1.97
	Unclassified Ty3/gypsy	8.92
rDNA		0.12
DNA Transposon		0.58
Satellite DNA		1.99
Unclassified		0.68
Total genome proportion		67.2

## Data Availability

The data presented in this study are openly available in Genbank (Bioproject PRJNA93409).

## Supplementary Materials

The following supporting information can be downloaded at: https://www.mdpi.com/article/10.3390/genes14020491/s1, Supplementary Table S1. Primers used for amplification and isolation of repetitive sequences.
