# Supplementary material for: Differential Repeat Accumulation in the Bimodal Karyotype of Agave L
Source: Genes (Basel). 2023 Feb 15;14(2):491. doi: 10.3390/genes14020491 (PMC9956584; doi:10.3390/genes14020491)
Supplement: Supplementary file 1 [file genes-14-00491-s001.zip › genes-2150291-supplementary.pdf]

Supplementary Table S1. *Primers* used for amplification and isolation of repetitive sequences

Primer name	Sequence (5’-3’)	Size (bp)	Ta (ºC)	GC (%)	Fragment (bp)
OgreCL39Ctg378F	TAGTGAATGTCTTTGGGCCTACC	23	62,9	47,83	548
OgreCL39Ctg378R	TGCTTCAGACACGAAGATCTTGA	23	61,1	43,48	
SIRECL22Ctg385F	TGCAAGAAGAAAACCCAAGTGAA	23	59,3	39,14	284
SIRECL22Ctg385R	AGGACATGCATCTTTTGACCATC	23	61,1	43,48	
TarCL121Ctg47F	GCTTGAGTCTCCTATTTTGTGGC	23	62,9	47,83	698
TarCL121Ctg47R	CACCCCCACATTTTGAGGTAATG	23	62,9	47,83	
ChromoCL32Ctg253F	AATCTCTGGTCCCAAAAGACTCC	23	62,9	47,83	498
ChromoCL32Ctg253R	GGCATTACCTCGTAGTCCTAAGG	23	64,7	52,17	
AthilaCL9Ctg216F	CTTTTGGTGATCCGAGCATTCTC	23	62,9	47,83	512
AthilaCL9Ctg216R	CTGTGCCAACTAAGACGAATGAC	23	62,9	47,83	
TorkCL10Ctg699F	AAACGACCTGTCTAATCCTGCAT	23	61,1	43,48	583
TorkCL10Ctg699R	TTTCAGATCAGGTGGAGACTTCG	23	62,9	47,83	
SatCL3Ctg1182F	CTAGATTGAAACCCGAAACAAGGT	24	61,8	41,67	171
SatCL3Ctg1182R	ACTTTCAACCCCAATTAGCCTGA	23	61,1	43,48	
